# Addressing social inequity through improving relational care: A social–ecological model based on the experiences of migrant women and midwives in South Wales

**DOI:** 10.1111/hex.13333

**Published:** 2021-08-01

**Authors:** Laura Goodwin, Aled Jones, Billie Hunter

**Affiliations:** ^1^ Faculty of Health and Applied Sciences University of the West of England Bristol UK; ^2^ School of Healthcare Sciences Cardiff University Cardiff UK

**Keywords:** conceptual model, culturally safe care, ethnic inequality, healthcare relationship

## Abstract

**Background:**

Migrant and ethnic inequalities in maternal and perinatal mortality persist across high‐income countries. Addressing social adversity and inequities across the childbirth trajectory cannot be left to chance and the good intentions of practitioners. Robust, evidence‐based tools designed to address inequity by enhancing both the quality of provision and the experience of care are needed.

**Methods:**

An inductive modelling approach was used to develop a new evidence‐based conceptual model of woman–midwife relationships, drawing on data from an ethnographic study of relationships between migrant Pakistani women and midwives, conducted between 2013 and 2016 in South Wales, UK. Key analytic themes from early data were translated into social–ecological concepts, and a model was developed to represent how these key themes interacted to influence the woman–midwife relationship.

**Results:**

Three key concepts influencing the woman–midwife relationship were developed from the three major themes of the underpinning research: (1) Healthcare System; (2) Culture and Religion; and (3) Family Relationships. Two additional weaving concepts appeared to act as a link between these three key concepts: (1) Authoritative Knowledge and (2) Communication of Information. Social and political factors were also considered as contextual factors within the model. A visual representation of this model was developed and presented.

**Conclusions:**

The model presented in this paper, along with future work to further test and refine it in other contexts, has the potential to impact on inequalities by facilitating future discussion on cultural issues, encouraging collaborative learning and knowledge production and providing a framework for future global midwifery practice, education and research.

**Patient or Public Contribution:**

At the outset of the underpinning research, a project involvement group was created to contribute to study design and conduct. This group consisted of the three authors, an Advocacy Officer at Race Equality First and an NHS Consultant Midwife. This group met regularly throughout the research process, and members were involved in discussions regarding ethical/cultural/social issues, recruitment methods, the creation of participant information materials, interpretation of data and the dissemination strategy. Ideas for the underpinning research were also discussed with members of the Pakistani community during community events and at meetings with staff from minority ethnic and migrant support charities (BAWSO, Race Equality First, The Mentor Ring). Local midwives contributed to study design through conversations during informal observations of antenatal appointments for asylum seekers and refugees.

## 1 BACKGROUND

In high‐income countries, migrant and ethnic inequalities in pregnancy outcomes persist. In the United States, for example, there is an increasing mortality gap between non‐Hispanic Black and all other women,^1^, ^2^ where recent data show that Black women are at over three times higher risk of dying from pregnancy complications than White women.^1^, ^2^ In the United Kingdom, the most recent Confidential Enquiry into Maternal Deaths and Morbidity^3^ reports that women from Black ethnic minority backgrounds are more than four times more likely to die as a result of complications in their pregnancy compared to White women. Similarly, women of mixed ethnicity are nearly twice as likely and women from Asian backgrounds have a threefold risk of dying as a result of complications.

Ethnic inequalities also exist in perinatal outcomes, where UK mortality rates remain exceptionally high for babies of Black and Black British ethnicity: Stillbirth rates are over twice those for babies of White ethnicity and neonatal mortality rates are 45% higher.^4^ For babies of Asian and Asian British ethnicities, stillbirth and neonatal mortality rates are both around 60% higher than for babies of White ethnicity.^4^ Migrant women and babies are also at increased risk of mortality and morbidity; nearly a quarter of maternal deaths between 2015 and 2017 were women born outside the United Kingdom,^3^ and UK mortality reports call for ‘continued focus on action to address these disparities’^3^ (p. 5), while highlighting the role of midwifery in addressing inequities during pregnancy, birth and the early postnatal period.

One critical factor in midwifery care is the quality of the woman–midwife relationship, with extensive literature suggesting that these relationships significantly impact on women's experiences of care as well as pregnancy outcomes.^5^, ^6^, ^7^, ^8^, ^9^, ^10^ Indeed, the 2011 UK Confidential Enquiry into Maternal Deaths and Morbidity suggests that emotional support and effective communication were critical for the prevention of maternal mortality.^10^ More recently, the 2016 National Maternity Review by NHS England, ‘Better Births’^11^ found that women emphasized the importance of forming a relationship with the professionals caring for them, as this could enable midwives to better meet their needs, identify problems and provide a safer service.

The importance of the woman–midwife relationship is especially apparent in the literature on the experiences of migrant women and Black and Minority Ethnic (BME) women, where both parties consistently report lower maternity care satisfaction^12^, ^13^, ^14^ and less choice in their maternity care^12^, ^15^ than their White native counterparts. Research also suggests that midwives may have more difficulty forming relationships with both migrant and minority ethnic women,^16^, ^17^, ^18^ for example, due to language barriers or cultural differences, which can lead to negative stereotyping of women by midwives.^16^, ^18^ This can impact on women's help‐seeking behaviours^18^ and hinder women and midwives from establishing a ‘partnership approach’ to care, as promoted by the UK model of midwifery.^19^

Previous research and healthcare policies have focused on staff training initiatives, such as cultural competence or awareness training, to promote culturally safe and congruent maternity care. While the concept of cultural competence continues as the foremost approach to addressing diversity in healthcare, it has important conceptual limitations,^20^ and is argued to be overgeneralising, simplistic and impractical.^21^ The dominance of this limited approach to understanding diversity exists as a result of the lack of robust, evidence‐based tools informed by the experiences of both service users and providers. Such evidence‐based tools, which draw on the experiences of service users and care providers, could be used in education and practice settings to address inequity by increasing mutual understanding, thus enhancing both the quality of care provision and the experience of care.

This gap in understanding and the clear need to create evidence‐based tools designed for practical application influenced the authors to develop an original model of woman–midwife relationships, informed by our ethnographic study of relationships between migrant Pakistani women and UK midwives in South Wales^22^ (described in full in an earlier issue of this journal). The remainder of this paper describes the development of this new social–ecological model of woman–midwife relationships, which conceptualizes not only interpersonal but also social and ecological factors that may serve as barriers or facilitators to a positive woman–midwife partnership approach to maternity care. Through the visual representation of the model, we aim to present the relationship between a woman and her midwife in a dyadic and holistic way that reflects the experiences of those receiving and those providing the care. By doing so, our intention is to encourage better informed and culturally aware/sensitive ways of providing services to ethnic minority women in the United Kingdom to address social inequities during pregnancy, birth and postnatally.

## 2 METHODS

The development of the inductive model was undertaken in three phases, which are described in the following sections: (i) Phase 1: the ‘underpinning research’ (an ethnographic study of relationships between migrant Pakistani women and midwives, conducted between 2013 and 2016 in South Wales, UK^22^); (ii) Phase 2: searching for congruence with existing models of healthcare relationships; and (iii) Phase 3: creating a new social–ecological model of woman–midwife relationships.

### 2.1 Phase 1: The underpinning research informing the model's development

Data used to develop the model presented in this paper were generated by an ethnographic study that took place in the South Wales region of the publicly funded UK National Health Service (NHS). The ethnographic data consisted of semi‐structured interviews with midwives and pregnant migrant Pakistani women, observations of antenatal appointments, community immersion and a review of relevant media.

Participants in interviews and observations included 11 NHS midwives working in the industrialized South Wales region of the United Kingdom; seven first‐generation migrant Pakistani women who were between 3 and 6 months pregnant and receiving NHS maternity care in the same region; one migrant Pakistani woman who was the mother of another participant; and one language interpreter (female) who had also migrated to the United Kingdom from Pakistan and experienced the UK maternity system. Length of residency in the United Kingdom ranged from 2 to 15 years, with a mean length of residency of 7 years. The focus was on migrant Pakistani women specifically, as at the time of study design, Pakistan was the second most common country of birth for non‐UK‐born mothers,^23^ and Pakistani women were at significantly increased risk of infant maternal mortality when compared to all other ethnic groups in the United Kingdom.^10^

The review of relevant media involved searching for news stories, healthcare policies, social media posts and government legislation relating to immigration, maternity care, ethnic inequalities and healthcare provision. Searches were carried out under the ‘news’ and ‘scholar’ advanced options of an internet search engine, using keywords such as ‘migrants’, ‘midwifery’, ‘inequalities’ and ‘policy’. Additionally, hardcopies of local papers were skimmed for relevant stories on a daily basis, and government and health board websites were searched for relevant policies every 6 months. Social media posts were also scanned on a frequent basis.

Community immersion involved the lead author volunteering for a number of charities providing support and advice to minority ethnic and migrant individuals (BAWSO, Race Equality First, The Mentor Ring) and attending community events to raise awareness for BME and migrant health issues, which led to around 30 h of working and socializing with members of the Pakistani community. This provided opportunities for the lead author to familiarize herself with the Pakistani culture, provided invaluable information for the design and early stages of the underpinning research and facilitated the involvement of stakeholders. Immersion in the midwifery setting involved informal observations of antenatal appointments for asylum seekers and refugees, attendance at midwifery conferences and shadowing midwives to learn about the day‐to‐day practice of midwifery.

In addition to this fieldwork, a Project Involvement Group was created. This group consisted of the three authors, an Advocacy Officer at Race Equality First and an NHS Consultant Midwife. This group met regularly throughout the research process, and members were involved in discussions regarding ethical/cultural/social issues, recruitment methods and the creation of participant information materials.

Findings from this study highlighted the complexity of relationships between women and midwives, and suggested a number of influential nested and interrelated themes. These included the role of family relationships; participants' relationships with culture and religion; understanding of different healthcare systems; attitudes towards authoritative knowledge; and perceived function of communication of information.^22^ A full description of the study methods and key themes can be found in the findings paper, published in a previous volume of this journal.^22^

### 2.2 Phase 2: Exploring existing models of woman–midwife relationships

In the next stage, we began by comparing early data from our underpinning research with existing models of healthcare relationships to explore the ways in which our themes, later named as concepts, aligned and differed. As is common in the healthcare literature, conceptual models are developed from research in an embryonic fashion, where key themes developed from the data are translated into model ‘components’ or ‘concepts’. Conceptual models of midwifery provide different ways of looking at practice and alternative ways of working with women and their families.^24^ They can also provide a framework for organizing education and identifying research questions.^24^

Early on in this process, we realized that the influence of social and political contextual factors on the woman–midwife relationship was reminiscent of a theory of child development, namely, the ‘Ecological Systems Theory’^25^ (EST) developed by Bronfenbrenner. EST proposes that child development should be viewed from a social–ecological perspective, placing the child in the centre of a ‘layered’ system, with all layers interacting and influencing development. These layered systems range from the child's individual interactions to the social and political context in which the child is raised. To correctly study human development, Bronfenbrenner argues that one has to see within, beyond and ‘across’ how these systems interact. The emphasis on systems interaction resonated with our ethnographic findings, as woman–midwife relationships were influenced by a number of social and ecological factors (our themes) that could not be studied in isolation.

We then discovered that the EST theory had previously been applied to models of healthcare relationships. For example, Hummell and Gates^26^ built on the systems approach, suggesting that healthcare relationships operate within a series of ‘nested dimensions’. The concept of nested dimensions also resonated with our findings, suggesting that to understand the quality of healthcare relationships and their impact on women's experiences and outcomes, it is crucial to acknowledge the complexities of interacting systems that might influence these relationships. A model proposed by Higgs^27^ similarly posits that each person exists within a network of multiple relationships, which variously impact on their encounters with healthcare professionals. Although relevant to our emergent research findings, these existing models focus on each person's individual factors such as knowledge, attitudes and beliefs separately, thus neglecting the interactions between, in this case, the woman and the midwife's own social–ecological influences. In short, we felt that existing models were not sufficiently sensitive to the interaction and nested complexities occurring between women and midwives that we were identifying in the data. We therefore began the process of creating a new conceptual framework to better understand our data and capture the relational complexities of partnership working therein.

### 2.3 Phase 3: Creating a new social–ecological model of woman–midwife relationships

The process of developing a new model was undertaken in parallel with data collection and analysis. For example, key analytic themes identified in the data were translated into social–ecological concepts (Table 1). The lead author produced a number of prototype models to visually represent how the socio–ecological concepts appeared to interact (i.e., how the social–ecological influences on the woman interacted with the social–ecological influences on the midwife). Each iteration of the prototype model was discussed with the coauthors for refinement and agreement and, in turn, informed further analysis of transcripts, observations and field notes. The model continued to be refined until all data had been analysed and there was consensus amongst the authors. The process of creating the model can, therefore, be described as both inductive and iterative.

**Table 1:** Illustrative examples of the analytic themes from the underpinning research, displayed alongside the corresponding theoretical concepts of the newly developed social–ecological model of woman–midwife relationships

Example quote from data	Analytic theme	Theoretical concept
‘Whenever we have a baby, we follow our elders. Our grandparents, our mother‐in‐law, our mothers—we follow them. We don't try to follow what the midwife wants to say to us—what the midwife is saying for safety. We don't bother—frankly speaking we don't bother…We follow our grandparents, our mother‐in‐law and our mothers’.	Women's relationships with mothers/mothers‐in‐law	Family relationships (woman)
‘Some things are related to our religion so it should be ok. For [midwives] as well. Because we have to shave our children's head. So it's religious. You have to weigh it. So they will accept this. We have to do circumcision for the boys. It's important in our religion. So they should be ok with it’.	Traditional Pakistani maternity practices	Culture and religion (woman)
‘They just go [to the doctor], straight away [in Pakistan]. Take a number and sit. And whenever they call them—they go and tell the doctor what's going on. But if you go in a very good medical centre [in Pakistan] there are [only] a couple of people [waiting]—that's why it's not very busy. So whenever you go, straight away you see the doctor. And that's why people don't know about appointments, you know, to make them [in the UK]’.	Understanding different healthcare systems	Healthcare system (woman)
‘They're inclined to talk for them as well… You're not quite knowing what the lady herself is thinking. I think mothers‐in‐law can be quite, the dominant relative. So they're inclined to, the mother‐in‐law, if she comes, to sort of dominate the consultation’.	The involvement of mothers‐in‐law	Family relationships (midwife)
‘Pregnant women shouldn't fast. And I always find that if they are fasting then I'm kind of lecturing them “no—you shouldn't be fasting” and that kind of thing, and they do get a bit funny about it. Because they want to do it, and I'm saying no you shouldn't do it. And that can cause a bit of—you can see that they're not happy that I'm saying no you shouldn't’.	Cultural practices	Culture and religion (midwife)
‘The traditions at home are completely different and how many visits they get—some are quite surprised at how many they get and some are surprised that they're not getting more. So yeah—it is different…You know—the whole maternity system at home’.	Understanding different healthcare systems	Healthcare system (midwife)
‘I would listen to the midwife. Because she's obviously the person who's more experienced in that. But then it's tradition… and you kind of respect tradition as well. I don't know—it's a bit difficult. How would you balance it?’	Authoritative knowledge	Authoritative knowledge
‘What I dislike is people who come in and they've got a list of demands. “You need to write me a letter for housing. You need to do this—you need to do that” That's all they want! Care isn't always a priority for them…they'll only come when they want something’.	Perceived purpose of communication/appointments	Communication of information

## 3 RESULTS: THE MODEL

**Figure 1:**
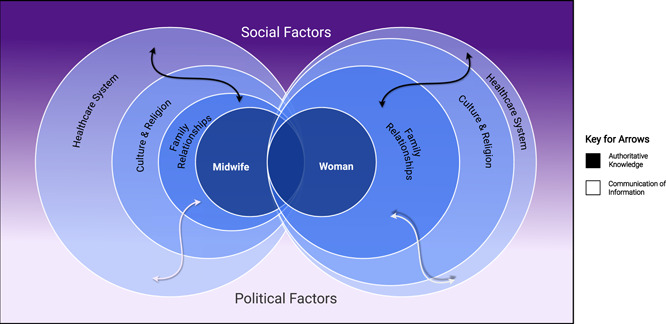
A social–ecological model of woman–midwife relationships

In this paper describing evidence‐based model development, the results are in fact the model itself. A visual representation of the newly developed social–ecological model of woman–midwife relationships can be seen in Figure 1.

In this visual representation, different widths of circles represent the relative importance (or ‘weighting’) of each of the three key concepts for each participant group; the wider the circle, the more influence that concept appeared to have for that participant group. The white and black arrows represent two concepts that originally emerged as ‘weaving themes’ from the data: ‘authoritative knowledge’ and ‘communication of information’. The purple and white background symbolizes the way in which data relating to the main social–ecological relationship concepts were situated within the social and political issues of public perception of immigration and reviews of failing UK maternity services. The gradient effect represents overlaps between these social and political issues.

The following sections further unpack the model by explaining how the various elements of the model dynamically interact, with direct reference to our previously published research findings.^22^

### 3.1 The three key concepts

Three key concepts were developed from the three key themes and findings of the underpinning research: (1) the Healthcare System; (2) Culture and Religion; and (3) Family Relationships. From our original findings, it was clear that participants placed different ‘weighting’ on the importance of these concepts in terms of their influence on the woman–midwife relationship.^22^ For example, the ‘Healthcare System’ was the most commonly discussed concept by midwives when interviewed about their relationships with women,^22^ where the biggest source of tension in their relationships with migrant Pakistani women was perceived to result from women's late arrival, or nonattendance, at NHS antenatal appointments.

In contrast, the ‘Healthcare System’ was discussed less frequently by migrant Pakistani women.^22^ Instead, women mostly focused on the impact of ‘Family Relationships’ and ‘Culture and Religion’ on their relationships with midwives.^22^ It was therefore important to demonstrate the different weightings of these concepts (achieved by varying the width of the circles on the diagrammatic representation of the model) and how they interacted to influence the woman–midwife relationship.

### 3.2 Weaving concepts

The two ‘weaving concepts’ were developed from the ‘weaving themes’ identified from the original study data.^22^ These concepts, ‘authoritative knowledge’ and ‘communication of information’, act as links between all other concepts within the social–ecological model.

In the underpinning research, authoritative knowledge (i.e., whose ‘knowledge’ carried most weight) was apparent in instances where competing sources of knowledge sometimes disrupted midwife–woman relationships. For example, women's relationships with midwives would, on occasion, exist within the context of potentially incongruent advice and knowledge regarding the baby's appearance and well‐being. For example, while women's female relatives encouraged cultural practices such as eyeliner or glass bracelets on babies, midwives raised concerns over the safety of these cultural practices.^22^ Such incongruence could result in relational difficulties, and the model helps to capture the significance and weight that family relationships and cultural dynamics bring to bear on women's relationships with midwives.

Communication of information affected relationships where the perceived purpose of communication differed. For example, while midwives were observed to attempt to build close relationships with women during antenatal appointments through social chat and humour, women often appeared to reject these exchanges in favour of more transactional encounters where medical testing and information provision were the focus of the consultation. Midwives reported that transactional exchanges such as requests for letters or signatures for other agencies (i.e., housing support) led to feelings of frustration, and many felt that they had been ‘used’ by women in such instances.

### 3.3 Contextual factors

The social and political contextual factors within which the woman–midwife relationship unfolded, depicted as the merging purple into the white background in Figure 1, were developed from a review of relevant media, carried out as part of the underpinning research. At the time of this underpinning research, immigration featured significantly in UK media reports, with an overwhelming negative tone towards migrants focussing on the perceived need to restrict and reduce population inflow. Public opinion polls reflected this media attention, with a survey by The Migration Observatory^28^ reporting that a large majority of people in Britain felt that there were too many migrants in the United Kingdom, fewer migrants should be given UK residency and legal restrictions on immigration should be tighter. At the same time, UK maternity services were also under scrutiny, as a number of media reports highlighted failings in care.^29^, ^30^ Interestingly, the topics of migration/population controls and significant concerns about the quality and safety of maternity care have continued to feature prominently in the UK news media.

Despite no direct reference to these contextual factors during interviews and observations, it was important to acknowledge these broader contextual factors when considering interactions between migrant Pakistani women and UK midwives during the study period and when developing a model of these relationships. Awareness of hostile social attitudes may have resulted in migrant Pakistani women participants feeling unable to voice concerns about their care, or media reports of NHS care failures may have resulted in doubts about the quality of UK healthcare provision. Such contextual conditions could be shared by the midwife participants. Their attitudes towards these factors may have impacted upon their relationships with women adversely, if negative views on immigration were shared, or positively, if increased attempts were made to build relationships with women and work in partnership to dispel beliefs about poor care and inequity of care.

## 4 DISCUSSION

Migrant and ethnic inequalities in maternal and perinatal mortality persist across high‐income countries,^1^, ^2^, ^3^, ^4^ and the factors contributing to vulnerabilities are complex and multi‐faceted.^31^ To offer safe care that is of high quality, individualized and culturally sensitive, maternity care providers must acknowledge and value diversity among service users,^5^ in addition to reflecting on the social and ecological influences that they themselves bring to relationships. Establishing common goals and mutual willingness to understand each other's perspectives all have a positive impact on health outcomes,^32^, ^33^ but can entail complex work. Addressing social inequities across the childbirth trajectory cannot be left to chance and the good intentions of healthcare professionals. What is needed are robust, evidence‐based tools designed to address inequity by enhancing both the quality of provision and the experience of care.^34^ In this paper, we present a new model of woman–midwife relationships, which offers a reflective and practical approach to establishing common goals, reducing assumptions and stereotyping and exploring and better understanding the perspectives of others to encourage a partnership model of care.

This model aligns with the four key concepts underpinning midwifery care theory^24^: Person (the Woman); Health (navigation of the Healthcare system); Environment (Culture and Religion; Social and Political factors); and Midwifery (the Midwife). It builds on previous models of healthcare relationships^25^, ^26^, ^27^ by considering not only the individual influences of social and ecological concepts but also the interactions between the two parties' social and ecological influences and how these may affect interpersonal relationships and partnership approaches to care. This new model of relationships also allows for visual representation of the relative importance of each concept in influencing this healthcare relationship, by depicting a ‘weighting’ of each concept and any incongruences, differences and/or similarities between women and midwives.

While models can provide a framework within which midwifery education and research can be better understood,^24^ they can also guide the way midwives work with women^35^ and offer an opportunity to improve practice. However, it is important to note that the use of conceptual models can unintentionally prevent individuals from approaching situations in an open way. The implementation of models of ‘cultural competence’, for example, has sometimes led to the legitimisation of cultural stereotypes.^36^

The model presented in this paper attempts to minimize this risk by emphasizing the individuality of relationships and encouraging actors to reflect on how the diagrammatic representation of the conceptual model should be adapted to fit their own specific relationships, rather than generalizing solutions to all relationships. Indeed, a strength of our model is that it aligns with the idea of cultural ‘humility’, rather than that of cultural ‘competence’. The humility approach encourages healthcare professionals to continually engage in self‐reflection and self‐critique, rather than attempting to ‘know’ about the culture of the ‘other’.^37^ As such, cultural humility addresses many of the critiques of cultural competency models. For example, the cultural humility approach explicitly acknowledges power differences between healthcare providers and service users^20^ and advocates for practitioner self‐reflection on what bias and assumptions they may bring to the provider–client relationship when working with people from different backgrounds.^20^ Awareness of this power imbalance is especially important for migrant service users who may already feel at a disadvantage due to issues with navigating the healthcare system or accessing the appropriate care.^22^

### 4.1 Implications for practice

It is proposed that this model be used as a tool to encourage healthcare staff to reflect on how their beliefs, assumptions and values may influence their relationships with women. The concept and visual representation of differential weighting could be used to facilitate understanding of the dynamic complexities of the woman–midwife relationship and the challenges that authentic partnership working present. By visually highlighting potential differences in healthcare, cultural and social priorities, as well as differences in the expectations and experiences of midwives and the women they care for, potential misalignments in priorities and/or knowledge could be identified and openly addressed, rather than both parties operating on the basis of differing and unstated expectations and norms.

For example, while navigation of the healthcare system (i.e., attending all appointments and arriving on time) was emphasized by midwives as a key factor in their relationships with women, this was not a common theme in conversations with women. By using the model to identify that women may be unaware that they are not navigating the NHS system ‘effectively’, and that this is causing their midwife to view their relationship negatively, it may be possible to address and align expectations and improve relationships. Improvement of these relationships may, in turn, promote more of a partnership approach to maternity care.

Although the model presented in this paper was developed from a study of migrant women,^22^ many of the model's themes and principles could be applied to all woman–midwife interactions. However, it is important to note the existence of individual differences in terms of expectations and priorities and to acknowledge that some pairings of women and midwives will converge more closely on these factors than others. Therefore, it is our recommendation that expectations of UK maternity care are addressed and managed not only at a group level (i.e., all women and midwives) but also on an individual level (i.e., exploring and managing expectations for each individual pairing of woman and midwife). By acknowledging how their relationships with women may be negatively impacted, we hope that practitioners may take steps to avoid this happening, therefore improving relationships, increasing understanding and ultimately contributing to a reduction in inequalities.

In addition to self‐reflection, we propose using the model's diagrammatic representation to facilitate discussions between women and midwives on these issues. Each party could personalize the social–ecological model according to their own weighting of concepts underpinned by their values and beliefs, thus exposing otherwise unsaid or assumed, yet potentially critical information. Thus, care experiences for women could be enhanced, in addition to improving work experiences for midwives. Discussions regarding the differences in values and beliefs may help to reduce stereotyping, identify social inequities and facilitate a better understanding of the complex factors that come into play during maternity care. Such discussions may also lead to better recognition of information needs, for example, maternity care staff may recognize that better education about the configuration of UK maternity systems and clearer information about care delivery need to be provided to women.^5^ This would then allow for more individualized care, and has the potential to impact on social inequalities. Using the model as a script for dialogue would, however, require sensitive awareness of the inherent power differentials in the woman–midwife relationship, whereby the midwife represents institutional power and authority.

Further work to develop, deploy and test this model could include the development of culturally appropriate ‘option grids’ to enhance equal participation in decision‐making. Option grids are decision support tools that work by presenting common patient‐generated questions (e.g., what are the chances of miscarriage from an amniocentesis test) against concise evidence‐based answers.^38^ The grid format provides a simple comparison of options allowing service users to work through key questions and answers, comparing their options, highlighting which issues matter most to them and discussing these key questions in more detail with their healthcare provider. Using our model, new option grids could be developed to facilitate discussions between midwives and BME women about traditional cultural practices during pregnancy, providing evidence for the safety of these practices alongside women's beliefs and thoughts regarding the cultural value of these traditions. The popularity of option grids appears to be growing,^39^ as they demonstrate respect for service users' views, while also providing healthcare providers with an objective, nonconfrontational way of approaching and recording potential safety concerns. Documented use of such grids can also provide evidence of midwives' information‐sharing and so may reduce anxieties around professional accountability.^39^

### 4.2 Implications for education

There is growing emphasis on the critical role played by relational skills in midwifery. The 2019 World Health Organization Framework for Midwifery Education Quality^40^ suggests that education should enable midwives to learn how to communicate, build relationships and understand and respect cultural differences and context. However, learning in academic settings is not always sufficient to sustain these understandings and capabilities in practice,^27^ and so alternative methods of cultural learning, such as multimedia approaches, are necessary to accommodate multiple learning styles and encourage self‐reflection.

The diagrammatic representation of the model presented in this paper could therefore be developed as a multimedia teaching resource for student midwives to foster awareness of the complexities of woman–midwife relationships. The model's grounding in the realities of clinical practice should make it more acceptable to practitioners and provide student midwives with the opportunity to explore their own attitudes towards differing social and cultural values in the protected setting of the learning environment. Students could personalize and reflect on their own models using technologies ranging from computer software to pen and paper, altering the width of circles to visually represent how their values and beliefs sit within each socio–ecological dimension and how this may affect their relationships with women who hold differing values and beliefs (i.e., where the width of their own circles varies greatly from those of their imaginary service user). Pregnant women from different ethnicities and cultures could be invited into guest lectures or seminars to contribute to the same activity, creating opportunity for experiential learning and coproduction of knowledge. This teaching resource could then be used during midwifery placements to bridge the classroom–practice gap and improve equity in pregnancy experiences through service design.

### 4.3 Strengths and limitations

This study builds on previous models of healthcare relationships to develop a robust evidence‐based tool to support practice. The model was derived from data collected from observations and interviews with Pakistani women with recent migrant status, living in a particular South Wales,^22^ and has yet to be tested with other groups. However, the principles enshrined can be applied to other contexts and situations, and as such, this model has the potential for transferability to other settings where similar inequities exist. For example, situations where inequalities, discrimination and/or stereotyping impact on woman–midwife relationships (i.e., social class or urban/rural divides). Further testing and refinement of the model would be needed to test its utility in diverse settings and to indicate how services could be adapted to enhance a partnership approach to care and decision‐making.

## 5 CONCLUSIONS

This paper presents a new social–ecological model of woman–midwife relationships, based on findings from an ethnographic study of migrant Pakistani women and midwives in the United Kingdom. The diagrammatic representation of this model provides a valuable visualisation of the complexities of partnership approaches to care and offers a window into possible reasons for tension and disconnect, together with the means for addressing them. The creation of such a model is timely, as migrant and ethnic inequalities in maternal and perinatal mortality persist across high‐income countries.^1^, ^2^, ^3^, ^4^

This model, along with future work to further test and refine it in other contexts, has the potential to address certain aspects of inequity by facilitating future discussion on cultural issues, encouraging collaborative learning and knowledge production and providing a framework for future global midwifery practice, education and research that has equitable partnerships at its heart.

## CONFLICT OF INTERESTS

The authors declare no conflict of interests.

## Data Availability

Data sharing is not applicable to this article as no new data were created or analysed in this study.

## References

[1] Creanga AA , Syverson C , Seed K , Callaghan WM . Pregnancy‐related mortality in the United States, 2011‐2013. Obstet Gynecol. 2017;130(2):366‐373. 28697109 10.1097/AOG.0000000000002114 PMC5744583

[2] Petersen EE , Davis NL , Goodman D , et al. Vital signs: pregnancy‐related deaths, United States, 2011‐2015, and strategies for prevention, 13 states, 2013‐2017. MMWR Morb Mortal Wkly Rep. 2019;68(18):423‐429. 31071074 10.15585/mmwr.mm6818e1 PMC6542194

[3] Knight M , Bunch K , Tuffnell D , et al. *Saving lives, improving mothers' care lessons learned to inform maternity care from the UK and Ireland confidential enquiries into maternal deaths and morbidity 2016‐18*. Oxford: National Perinatal Epidemiology Unit, University of Oxford; 2020.

[4] Draper ES , Gallimore ID , Smith LK , et al. *MBRRACE‐UK perinatal mortality surveillance report, UK perinatal deaths for births from January to December 2018*. Leicester: The Infant Mortality and Morbidity Studies, Department of Health Sciences, University of Leicester; 2020.

[5] Higginbottom GMA , Evans C , Morgan M , Bharj KK , Eldridge J , Hussain B . Experience of and access to maternity care in the UK by immigrant women: a narrative synthesis systematic review. BMJ Open. 2019;9(12):e029478. 10.1136/bmjopen-2019-029478 PMC6955508 31892643

[6] Healthcare Commission. *Women's experiences of maternity care in the NHS in England: key findings from a survey of NHS trusts carried out in 2007*. London: Commission for Healthcare Audit and Inspection; 2007.

[7] Sandall J , Soltani H , Gates S , Shennan A , Devane D . Midwife‐led continuity models versus other models of care for childbearing women. Cochrane Database Syst Rev. 2016;4(4):004667. 10.1002/14651858.CD004667.pub5 PMC8663203 27121907

[8] Jonkers M , Richters A , Zwart J , Öry F , van Roosmalen J . Severe maternal morbidity among immigrant women in the netherlands: patients' perspectives. Reprod Health Matters. 2011;19(37):144‐153. 21555095 10.1016/S0968-8080(11)37556-8

[9] Berg M . A midwifery model of care for childbearing women at high risk: genuine caring in caring for the genuine. J Perinat Educ. 2005;14(1):9‐21. 10.1624/105812405X23577 PMC1595225 17273417

[10] Cantwell R , Clutton‐Brock T , Cooper G , et al. Saving mothers' lives: reviewing maternal deaths to make motherhood safer: 2006‐2008. The eighth report of the confidential enquiries into maternal deaths in the United Kingdom. BJOG. 2011;118(suppl 1):1‐203. 10.1111/j.1471-0528.2010.02847.x 21356004

[11] National Maternity Review . Better births: Improving outcomes of maternity services in England. *A Five Year Forward View for maternity care*. NHS England; 2016.

[12] Redshaw M , Rowe R , Hockley C , Brocklehurst P . Recorded Delivery: A National Survey of Women's Experience of Maternity Care 2006. National Perinatal Epidemiology Unit; 2007.

[13] Richens Y . Exploring the Experiences of Women of Pakistani Origin of UK Maternity Services. Department of Health; 2003.

[14] Small R , Roth C , Raval M , et al. Immigrant and non‐immigrant women's experiences of maternity care: a systematic and comparative review of studies in five countries. BMC Pregnancy Childbirth. 2014;14(1):152. 24773762 10.1186/1471-2393-14-152 PMC4108006

[15] Redshaw M , Heikkila K . Ethnic differences in women's worries about labour and birth. Ethn Health. 2011;16:213‐223. 21500115 10.1080/13557858.2011.561302

[16] Bowler I . ‘They're not the same as us’: midwives' stereotypes of South Asian descent maternity patients. Sociol Health Illn. 1993;15:157‐178.

[17] Essén B , Binder P , Johnsdotter S . An anthropological analysis of the perspectives of Somali women in the West and their obstetric care providers on caesarean birth. J Psychosom Obstet Gynaecol. 2011;32:10‐18. 21291343 10.3109/0167482X.2010.547966 PMC3055712

[18] Degni F , Suominen S , Essén B , El Ansari W , Vehvilainen‐Julkunen K . Communication and cultural issues in providing reproductive health care to immigrant women: health care providers' experiences in meeting Somali women living in Finland. J Immigr Minor Health. 2012;14:330‐343. 21465142 10.1007/s10903-011-9465-6

[19] Royal College of Midwives. *High Quality Midwifery Care. * 2014. Accessed August 25, 2016. https://www.rcm.org.uk/media/2354/high-quality-midwifery-care.pdf

[20] Beagan BL . A critique of cultural competence: assumptions, limitations, and alternatives. In: Frisby CL , O'Donohue WT , eds. Cultural Competence in Applied Psychology. Springer; 2018:123‐138.

[21] Shepherd SM . Cultural awareness workshops: limitations and practical consequences. BMC Med Educ. 2019;19(1):14. 30621665 10.1186/s12909-018-1450-5 PMC6325797

[22] Goodwin L , Hunter B , Jones A . The midwife–woman relationship in a South Wales community: experiences of midwives and migrant Pakistani women in early pregnancy. Health Expect. 2018;21(1):347‐357. 28960699 10.1111/hex.12629 PMC5750740

[23] Office for National Statistics. *Statistical Bulletin: Births in England and Wales by Parents' Country of Birth, 2014*. 2015. Accessed February 8, 2020. https://www.ons.gov.uk/peoplepopulationandcommunity/birthsdeathsandmarriages/livebirths/bulletins/parentscountryofbirthenglandandwales/2015-08-27

[24] Bryar R , Sinclair M . Conceptualizing midwifery. In: Bryar R , Sinclair M , eds. Theory for Midwifery Practice. Vol 2. Palgrave Macmillan; 2011:16.

[25] Bronfenbrenner U . The Ecology of Human Development: Experiments in Nature and Design. Harvard University Press; 1979.

[26] Hummell J , Gates A . Negotiating healthcare relationships through communication. Higgs J , Croker, A , Tasker, D , Hummell, J , Patton, N . Health practice relationships. Brill Sense, 2014:57‐64.

[27] Higgs J . Health practice relationships. In: Higgs J , Croker A , Tasker D , Hummell J , Patton N , eds. Health Practice Relationships. Brill Sense; 2014:1‐8.

[28] Blinder S . UK Public Opinion toward Migration: Determinants of Attitudes. Migration Observatory briefing. COMPAS, University of Oxford; 2011.

[29] Kirkup B . The Report of the Morecambe Bay Investigation: An Independent Investigation into the Management, Delivery and Outcomes of Care Provided by the Maternity and Neonatal Services at the University Hospitals of Morecambe Bay NHS Foundation Trust from January 2004 to June 2013. The Stationery Office; 2015.

[30] O'Neill O . Safe Births: Everybody's Business. An Independent Inquiry into the Safety of Maternity Services in England. King's Fund; 2008.

[31] Goodwin L , Hunter B , Jones A . Immigration and continuing inequalities in maternity outcomes: time to reexplore the client‐provider relationship? Int J Childbirth. 2015;5(1):12‐19.

[32] Ackerson LK , Viswanath K . The social context of interpersonal communication and health. J Health Commun. 2009;14(S1):5‐17. 19449264 10.1080/10810730902806836

[33] Duggan A . Understanding interpersonal communication processes across health contexts: advances in the last decade and challenges for the next decade. J Health Commun. 2006;11(1):93‐108. 10.1080/10810730500461125 16546921

[34] Tunçalp Ӧ , Were WM , MacLennan C , et al. Quality of care for pregnant women and newborns—the WHO vision. BJOG. 2015;122(8):1045‐1049. 25929823 10.1111/1471-0528.13451 PMC5029576

[35] Fawcett J . Contemporary Nursing Knowledge: Analysis and Evaluation of Nursing Models and Theories. Vol 2. F.A. Davies; 2005.

[36] Jenks AC . From “lists of traits” to “open‐mindedness”: emerging issues in cultural competence education. Cult Med Psychiatry. 2011;35(2):209‐235. 21560030 10.1007/s11013-011-9212-4

[37] Fisher‐Borne M , Montana Cain J , Martin SL . From mastery to accountability: cultural humility as an alternative to cultural competence. Soc Work Educ. 2015;34(2):165‐181.

[38] Beattie B , Thomas, G , Durand, M , Elwyn, G , Fisher, J , Delbarre, A . Helping parents decide about down syndrome screening: using an option grid as a facilitation tool. BJOG. 2013;120(1):42‐43.23841804

[39] Seal RP , Kynaston, J , Elwyn, G , Smith, PE . Using an option grid in shared decision making. Pract Neurol. 2014;14(1):54‐56. 24151338 10.1136/practneurol-2013-000666

[40] World Health Organization. Strengthening Quality Midwifery Education for Universal Health Coverage 2030: Framework for Action. World Health Organization; 2019.

